# How a Hospital Saves Premature Infants

**Published:** 1923-09

**Authors:** 


### HOW A HOSPITAL SAVES PREMATURE INFANTS.

### A MANCHESTER EXPERIMENT.

TO the ninth annual report of the Manchester Babies' Hospital, Dr. Catherine Chisholm, the Chairman of the Medical Committee, contributes a remarkably interesting " Memorandum on Nursing Mothers," in which she describes the results of an endeavour to reduce the high mortality among premature infants and those of very light weight. Five nursing mothers were installed in temporary accommodation, two at a time, for a period of six months from the spring of 1922. During the previous four and a half years eighteen premature infants had been admitted, and of these eleven had died, three had been removed practically moribund and only four had survived—that is, 22.2 per cent. The experiment was modelled largely on the Wet Nursing Directory of Boston. The mothers brought their infants into the Hospital, and these infants had the first claim on three feeds a day, the rest going to the Hospital. These women each do work valued by the Matron at about 10s. a week. They receive this sum together with their food and their children's supplementary feeds. The women have fitted well into hospital life. The yield of milk is 27 to 31 ozs. each a day, in addition to their own children's feeds. The milk is drawn off rather than fed directly to the children.

### The Results.

The experiment was so successful that the women were kept on beyond the experimental six months. The results of the first sixty-two cases who have benefited by the mother's milk are as follows:—Of seventeen infants, premature or of very poor development, all under 6 lb., five died. One was congenital jaundice, one was a Mongol, one a pyloric stenosis, one was removed by the mother, one died within thirty hours of admission. Eleven lived—that is, 64.7 per cent., as compared with the previous record of 22.2 per cent. Further, in January and February of 1923, two separate infections of influenza have been given to the infants, and though premature infants have been attacked they have not succumbed. This is remarkable as it is well known that premature infants almost invariably succumb to such infections. The milk was also used for acutely ill infants other than premature children. It was noted that often an acutely dyspeptic baby benefited when only two or three breast feeds a day could be spared for it. Of forty-five infants treated, nine received breast milk only in the last few hours as a last hope, so these may be set aside in considering the result of this type of feeding. Of the remaining thirty-six, all very urgent and severe cases, 82.2 per cent. lived and only 17.8 per cent. died. This is a lighter mortality than the average of all cases in any year in this Hospital. The general mortality rate of the Hospital in 1922 was 21.36 per cent., and if, says Dr. Chisholm, we reckon the year from March 1, 1922 (the month in which the nursing mothers were installed), to March 1, 1923, the mortality is as low as 19.5 per cent., despite the fact that we have had such a very large number of premature infants admitted.

